# Supplementary material for: The First Whole Genome Sequence and Methylation Profile of Gerronema lapidescens QL01
Source: J Fungi (Basel). 2025 Sep 1;11(9):647. doi: 10.3390/jof11090647 (PMC12470499; doi:10.3390/jof11090647)

The First Whole Genome Sequence and Methylation Profile of *Gerronema lapidescens* QL01

Yanming Qiao[1], Zhiyuan Jia[2], Yuying Liu[2, 3], Na Zhang[2], Chun Luo[1], Lina Meng[1], Yajie Cheng[1], Minglei Li[3], Xiuchao Xie[1, *], Jianzhao Qi[2, 3, *]

[1] Shaanxi Province Key Laboratory of Bio-Resources, Qinba State Key Laboratory of Biological Resources and Ecological Environment (Incubation), School of Biological Science and Engineering, Shaanxi University of Technology, Hanzhong 723000, China.
[2] Shaanxi Key Laboratory of Natural Products & Chemical Biology, College of Chemistry & Pharmacy, Northwest A&F University, Yangling 712100, China.
[3] Center of Edible Fungi, Northwest A&F University, Yangling 712100, China.
[*] Correspondence: qjz@nwafu.edu.cn (J.Q.); xiexiuchao@snut.edu.cn (X. X.).

Content

Table S1. Next-generation sequencing data of *Gerronema lapidescens* QL01 genome. 1
Table S2. Estimation of genome size of *Gerronema lapidescens* QL01. 2
Table S3. Statistical table of *Gerronema lapidescens* QL01 assembly results. 3
Table S4. Statistical table of the length of the assembly sequence of *Gerronema lapidescens* QL01. 4
Table S5. Statistics of BUSCO evaluation of *Gerronema lapidescens* QL01 genome. 5
Table S6. Genetic Information the Statistical Table of protein-coding genes. 6
Table S7. Statistics of non-coding RNA annotation results in *Gerronema lapidescens* QL01 genome. 7
Table S8. Statistics of *Gerronema lapidescens* QL01 repetitive sequence annotation results. .8
Table S9. Anonation statistics table of *Gerronema lapidescens* QL01. 9
Table S10. Statistics for SNP of *Gerronema lapidescens* QL01. 10
Table S11. Statistics for methylation of Gerronema lapidescens QL01. 11
Table S12. The source (URL) statistics for 31 Basidiomycota and an Ascomycete used to phylogenetic analysis..12
Table S13. The composition of CAZymes of 16 representative Basidiomycetes. 14
Table S14. The source (URL) statistics for 16 representative Basidiomycetes used to CAZymes analysis. 15
Table S15. Statistics for SSR of *Gerronema lapidescens* QL01 and related edible fungi.........17
Table S16. Terpene enzymes in *Gerronema lapidescens* QL01 genomes. 20
Table S17. Quantify CpG site counts per core gene..21
Figure S1. Kmer-Depth and Kmer Species-Frequency Distribution Plot of *Gerronema lapidescens* QL01. 23
Figure S2. GC content density distribution plot of *Gerronema lapidescens* QL01. 24
Figure S3. Sequencing depth density distribution plot of *Gerronema lapidescens* QL01......25

Table S1. Next-generation sequencing data of *Gerronema lapidescens* QL01 genome.

Item	*Gerronema lapidescens* QL01
Raw data	6,981,239,100 bp
Clean data	6,568,644,900 bp
Clean data Q20	98.50%
Clean data Q30	94.70%
Clean data GC	44.95%

Table S2. Estimation of genome size of *Gerronema lapidescens* QL01.

Item	*Gerronema lapidescens* QL01
kmer	17
K-mer number	5,867,989,444
K-mer Depth	64
Genome size	87.49 Mbp
Heterozygous Ratio	0.52%
Repeat	35.51%

Genome size was estimated using genomescope v2.0 (http://genomescope.org/genomescope2.0/) software.

Table S3. Statistical table of *Gerronema lapidescens* QL01 assembly results.

**Item**	raw data
Total Length (bp)	82,231,084
N50 length (bp)	7,148,669
GC Content (%)	46.03
N rate (%)	0
Total_number	23

Table S4. Statistical table of the length of the assembly sequence of *Gerronema lapidescens* QL01.

Chr	Length (bp)	Nrate
Chr1	7,784,957	0
Chr2	8,652,620	0
Chr3	7,002,442	0
Chr4	8,430,268	0
Chr5	6,807,731	0
Chr6	6,296,936	0
Chr7	6,825,524	0
Chr8	6,816,636	0
Chr9	8,227,654	0
Chr10	7,607,356	0
Chr11	7,148,669	0
Ctg1	34,950	0
Ctg3	61,497	0
Ctg4	54,038	0
Ctg5	65,423	0
Ctg6	55,189	0
Ctg7	67,127	0
Ctg8	41,384	0
Ctg10	45,553	0
Ctg11	38,392	0
Ctg12	41,922	0

Table S5. Statistics of BUSCO evaluation of *Gerronema lapidescens* QL01 genome.

Gerronema lapidescens QL01	Assembly No.	Assembly ratio(%)	Annotation No.	Annotation ratio(%)
Complete BUSCOs (C)	714	94.1	708	93.4
Complete and single-copy BUSCOs (S)	700	92.3	690	91.0
Complete and duplicated BUSCOs (D)	14	1.8	18	2.4
Fragmented BUSCOs (F)	12	1.6	9	1.2
Missing BUSCOs (M)	32	4.2	41	5.4
Total BUSCO groups searched (n)	758	100.0	758	100.0

Single-copy for single-copy BUSCOs; duplicated for multicopy BUSCOs; Fragmented for fragmented BUSCOs; Missing for missing BUSCOs. The predicted genes were assessed for completeness using the BUSCO software (version: 4.1.4) based on the fungi database (fungi_odb10).

Table S6. Genetic Information the Statistical Table of protein-coding genes.

Item	*Gerronema lapidescens* QL01
Gene number	15,847
Average of gene length (bp)	2,361.46
Average of CDS length (bp)	1,498.04
Average of exon length (bp)	247.57
Average of exon number	6.05
Average of intron length (bp)	170.95

Table S7. Statistics of non-coding RNA annotation results in *Gerronema lapidescens* QL01 genome.

Class		number	Average length(bp)	Total length(bp)	% of genome
tRNA		193	84.63212	16,334	0.019864
snRNA tRNA	rRNA	129	3278.27907	422,898	0.514280
	18S	44	2197.31818	96,682	0.117574
	28S	42	7650.30952	321,313	0.390744
	5S	43	114.02326	4,903	0.005962
snRNA	snRNA	12	155.00000	1,860	0.002262
	CD-box	2	103.50000	207	0.000252
	splicing	10	165.30000	1,653	0.002010

rRNA is ribosomal RNA; tRNA is transport RNA; sRNA is small regulatory RNA; snRNA is nucleolar small RNA. **totalLen** and **meanLen** are the total length and mean length.

Table S8. Statistics of *Gerronema lapidescens* QL01 repetitive sequence annotation results.

Type	DNA	LINE	SINE	LTR	Other	Unknown	Total TE
**RepeatMasker TEs Length (bp)**	709,511	117,556	3,037	2,276,140	39	8,365	3,078,751
**RepeatMasker TEs % in genome**	0.86	0.14	0	2.77	0	0.01	3.74
**RepeatProteinMask TEs Length (bp)**	230,995	36,318	0	2,788,312	0	0	3,055,625
**RepeatProteinMask TEs % in genome**	0.28	0.04	0	3.39	0	0	3.72
**De novo Length (bp)**	9,469,184	1,035,707	145,227	21,416,465	0	1,671,796	32,752,898
**De novo % in genome**	11.52	1.26	0.18	26.04	0	2.03	39.83
**Combined TEs Length (bp)**	9,975,946	1,160,673	148,264	21,840,647	39	1,680,161	33,525,235
**Combined TEs % in genome**	12.13	1.41	0.18	26.56	0	2.04	40.77

Table S9. Anonation statistics table of *Gerronema lapidescens* QL01.

Item	Count	Percentage/%
Total	12,855	NA
Interproscan	7,198	55.99
GO	7,454	57.99
KEGG-ALL	10,594	82.41
KEGG-KO	3,484	27.10
Swissprot	5,851	45.52
TrEMBL	11,031	85.81
Nr	11,135	86.62
Annotated	11,188	87.03
Unannotated	1,667	12.97

Table S10. Statistics for SNP of *Gerronema lapidescens* QL01.

No.	Number
Chr1	88,651
Chr2	1,026,552
Chr3	816,925
Chr4	997,705
Chr5	807,981
Chr6	748,962
Chr7	383,031
Chr8	805,610
Chr9	995,212
Chr10	910,579
Chr11	863,473
Ctg1	98
Ctg2	516
Ctg3	4,032
Ctg4	1
Ctg5	1,269
Ctg6	1,161
Ctg7	615
Ctg8	1,915
Ctg9	3
Ctg10	5,741
Ctg11	275
Ctg12	1
Total	8,460,308

Table S11. Statistics for methylation of Gerronema lapidescens QL01.

No.	Number
Chr1	289,973
Chr2	315,130
Chr3	284,121
Chr4	347,592
Chr5	286,221
Chr6	243,792
Chr7	282,621
Chr8	260,099
Chr9	337,666
Chr10	324,188
Chr11	275,389
Ctg1	184
Ctg2	1,220
Ctg3	2,267
Ctg4	1,794
Ctg5	0
Ctg6	769
Ctg7	1,148
Ctg8	1,472
Ctg9	0
Ctg10	1,539
Ctg11	0
Ctg12	804
Total	3,258,016

Table S12. The source (URL) statistics for 31 Basidiomycota and an Ascomycete used to phylogenetic analysis.

Species	Source
Ustilago maydis	https://mycocosm.jgi.doe.gov/Ustma2_2/Ustma2_2.home.html
Rickenella mellea	https://www.ncbi.nlm.nih.gov/datasets/genome/GCA_004355085.1/
Trichaptum biforme	https://www.ncbi.nlm.nih.gov/datasets/genome/GCA_040954535.1/
Phellinidium pouzarii	https://www.ncbi.nlm.nih.gov/datasets/genome/GCA_004802695.1/
Sanghuangporus baumii	https://www.ncbi.nlm.nih.gov/datasets/genome/GCA_048932585.1/
Inonotus hispidus	https://www.ncbi.nlm.nih.gov/datasets/genome/GCA_024712875.1/
Stereum hirsutum	https://www.ncbi.nlm.nih.gov/datasets/genome/GCF_000264905.1/
Hericium rajendrae	https://www.ncbi.nlm.nih.gov/datasets/genome/GCA_033439255.1/
Hericium alpestre	https://www.ncbi.nlm.nih.gov/datasets/genome/GCA_004681135.1/
Gloeophyllum trabeum	https://www.ncbi.nlm.nih.gov/datasets/genome/GCF_000344685.1/
Cristinia sonorae	https://www.ncbi.nlm.nih.gov/datasets/genome/GCA_021029105.1/
Ganoderma sinense	https://www.ncbi.nlm.nih.gov/datasets/genome/GCA_002760635.1/
Gelatoporia subvermispora	https://www.ncbi.nlm.nih.gov/datasets/genome/GCA_000320605.2/
Wolfiporia cocos	https://www.ncbi.nlm.nih.gov/datasets/genome/GCA_029448655.1/
Laetiporus sulphureus	https://www.ncbi.nlm.nih.gov/datasets/genome/GCF_001632365.1/
Suillus brevipes	https://www.ncbi.nlm.nih.gov/datasets/genome/GCA_011800875.2/
Pleurotus ostreatus	https://www.ncbi.nlm.nih.gov/datasets/genome/GCF_014466165.1/
Pteruala gracilis	https://www.ncbi.nlm.nih.gov/datasets/genome/GCA_004369125.1/
Schizophyllum commune	https://www.ncbi.nlm.nih.gov/datasets/genome/GCF_000143185.2/
Gerronema lapidescens	This study
Lentinula edodes	https://www.ncbi.nlm.nih.gov/datasets/genome/GCF_021015755.1/
Dendrothele bispora	https://www.ncbi.nlm.nih.gov/datasets/genome/GCA_004369135.1/
Pluteus cervinus	https://www.ncbi.nlm.nih.gov/datasets/genome/GCA_004369065.1/
Amanita muscaria	https://www.ncbi.nlm.nih.gov/datasets/genome/GCA_000827485.1/
Tricholoma matsutake	https://www.ncbi.nlm.nih.gov/datasets/genome/GCA_026075535.2/

Species	Source
Lyophyllum atratum	https://www.ncbi.nlm.nih.gov/datasets/genome/GCA_014905825.1/
Psilocybe cubensis	https://www.ncbi.nlm.nih.gov/datasets/genome/GCF_017499595.1/
Cortinarius glaucopus	https://www.ncbi.nlm.nih.gov/datasets/genome/GCA_015039465.1/
Cyathus olla	https://www.ncbi.nlm.nih.gov/datasets/genome/GCA_036940585.1/
Agaricus bitorquis	https://www.ncbi.nlm.nih.gov/datasets/genome/GCA_030246685.1/
Agaricus bisporus	https://www.ncbi.nlm.nih.gov/datasets/genome/GCA_000300575.2/

Table S13. The composition of CAZymes of 16 representative Basidiomycetes.

Species	AA	CE	GH	GT	PL	CBM
Cyclocybe aegerita AAE3	112	34	222	65	18	29
Hypsizygus marmoreus 51987-8	123	24	225	73	22	25
Lentinula edodes Le(Bin) 0899 s	92	33	254	72	13	23
Lyophyllum decastes LRG-d1-5	125	37	246	71	23	34
Hymenopellis raphanipes CGG-A-s1	172	45	335	85	31	26
Pleurotus djamor MPG-05	258	48	398	90	47	106
Pleurotus eryngii ATCC 90797	171	30	234	69	35	27
Pleurotus ostreatus PC9	141	32	239	65	29	45
Stropharia rugosoannulata A15	149	39	222	70	15	24
Tricholoma matsutake 945	67	15	139	71	9	8
Volvariella volvacea WC 439	137	37	255	68	30	55
Mucidula mucida CBS558.79	70	27	125	7	17	6
Pleurotus cornucopiae ASM1967732	77	19	129	4	20	28
Pleurotus ostreatoroseus ASM52980	28	8	71	5	6	7
Gerronema lapidescens QL01	162	27	211	62	12	47
Cyathus olla SUT01	134	30	274	77	19	29

Table S14. The source (URL) statistics for 16 representative Basidiomycetes used to CAZymes analysis.

Species	Source
Cyclocybe aegerita AAE3	https://www.ncbi.nlm.nih.gov/datasets/genome/GCA_902728275.1/
Hypsizygus marmoreus 51987-8	https://www.ncbi.nlm.nih.gov/datasets/genome/GCA_001605315.2/
Lentinula edodes Le(Bin) 0899 ss1	https://www.ncbi.nlm.nih.gov/datasets/genome/GCF_021015755.1/
Lyophyllum decastes LRG-d1-5	https://www.ncbi.nlm.nih.gov/datasets/genome/GCA_026258425.1/
Hymenopellis raphanipes CGG-A-s1	https://www.ncbi.nlm.nih.gov/datasets/genome/GCA_036872995.1/
Pleurotus djamor MPG-05	https://www.ncbi.nlm.nih.gov/datasets/genome/GCA_029747585.1/
Pleurotus eryngii ATCC 90797	https://www.ncbi.nlm.nih.gov/datasets/genome/GCA_015484515.1/
Pleurotus ostreatus PC9	https://www.ncbi.nlm.nih.gov/datasets/genome/GCF_014466165.1/
Stropharia rugosoannulata A15	https://www.ncbi.nlm.nih.gov/datasets/genome/GCA_036873085.1/
Tricholoma matsutake 945	https://www.ncbi.nlm.nih.gov/datasets/genome/GCA_014904895.1/
Volvariella volvacea WC 439	https://www.ncbi.nlm.nih.gov/datasets/genome/GCA_001691835.3/
Mucidula mucida CBS558.79	https://www.ncbi.nlm.nih.gov/datasets/genome/GCA_015501055.1/
Pleurotus cornucopiae ASM1967732	
Pleurotus ostreatoroseus ASM52980	
Gerronema lapidescens QL01	This study
Cyathus olla SUT01	

Table S15. Statistics for SSR of *Gerronema lapidescens* QL01 and related edible fungi.

Species	Motif	No.	Percentage (%)	Length overall (bp)	Average Length (bp)	No. motifs type	Longest pattern
Wolfiporia cocos	Monomer	138	8.86%	2096	15.2	4	(C)$_{34}$
	Dimer	563	36.16%	8674	15.4	12	(AG)$_{63}$
	Trimer	707	45.41%	12783	18.1	53	(GAA)$_{23}$
	Tetramer	83	5.33%	1728	20.8	19	(TCAA)$_{10}$
	Pentamer	16	1.03%	570	35.6	15	(GCCGA)$_{26}$
	Hexamer	50	3.21%	1740	34.8	39	(CAGGTT)$_{10}$
	all SSRs	1557	100.00%	27591	17.7	142	(AG)$_{63}$
Megacollybia marginata	Monomer	433	12.41%	6304	14.6	4	(A)$_{38}$
	Dimer	985	28.23%	14800	15	11	(CA)$_{22}$
	Trimer	1716	49.18%	30216	17.6	59	(TAT)$_{19}$
	Tetramer	236	6.76%	5152	21.8	57	(GATG)$_{9}$
	Pentamer	71	2.03%	1920	27	34	(TTTCA)$_{12}$
	Hexamer	48	1.38%	1584	33	47	(CATGAC)$_{8}$
	all SSRs	3489	100.00%	59976	17.2	212	(A)$_{38}$
Megacollybia platyphylla	Monomer	1057	27.50%	18720	17.7	4	(AG)$_{31}$， (AG)31
	Dimer	709	18.44%	10694	15.1	11	(CT)$_{15}$,(AG)$_{15}$,(GT)$_{15}$, (TG)$_{15}$,(TC)$_{15}$,(AT)$_{15}$, (AT)$_{15}$,(TA)$_{15}$,(TC)$_{15}$, (AC)$_{15}$,(AG)$_{15}$,(CA)$_{15}$, (AT)$_{15}$,(TA)$_{15}$,(AT)$_{15}$, (TG)$_{15}$,(TG)$_{15}$,(TC)$_{15}$, (TC)$_{15}$,(AC)$_{15}$,(AG)$_{15}$, (GA)$_{15}$
	Trimer	1611	41.91%	29271	18.2	59	(CTA)$_{11}$
	Tetramer	298	7.75%	6440	21.6	52	(GAAG)$_{7}$,(TCCT)$_{7}$,(AAGG)$_{7}$,

Species	Motif	No.	Percentage (%)	Length overall (bp)	Average Length (bp)	No. motifs type	Longest pattern
							(GGAA)7,(CTTC)7,(GGAA)7, (CCGA)7,(AAGG)7,(CTTC)7, (TCCT)7,(AAGG)7,(AGGA)7, (AGGA)7,(TTCC)7,(GAAG)7, (ATAG)7,(AAGG)7,(AATC)7, (GGAA)7,(GAAG)7,(CTTC)7, (CTTC)7,(CTTC)7,(GGAA)7, (GGAA)7,(CTTC)7,(CTTC)7
	Pentamer	63	1.64%	1650	26.2	28	(GGTTA)6,(ATCCT)6, (TAATG)6,(ATTTA)6, (TAGGA)6,(ATAAA)6, (AGGAT)6,(GGATA)6, (TTTAA)6,(ATTTA)6, (TAAAT)6,(AAATT)6, (TTTAA)6,(GGTTA)6, (TCGTA)6
	Hexamer	106	2.76%	3234	30.5	72	(CGAAAC)6,(TATACA)6, (CAAGGA)6,(GCTTTG)6, (CAGCAC)6,(TGAAGG)6, (CAAGGT)6,(ATGAAA)6
	all SSRs	3844	100.00%	70009	18.2	226	(AG)31(AG)31
Gerronema lapidescens	Monomer	405	12.35%	6205	15.3	4	(T)39
	Dimer	1139	34.74%	16542	14.5	12	(AC)23
	Trimer	1521	46.39%	30777	20.2	60	(TAT)52
	Tetramer	131	4.00%	2792	21.3	35	(CCTT)9,(ATCT)9,(GAAG)9
	Pentamer	12	0.37%	315	26.3	9	(TATAT)7

Species	Motif	No.	Percentage (%)	Length overall (bp)	Average Length (bp)	No. motifs type	Longest pattern
	Hexamer	71	2.17%	3000	42.3	35	$(TTAGGG)_{21}$
	all SSRs	3279	100.00%	59631	18.2	155	$(TAT)_{52}$

Table S16. Terpene enzymes in *Gerronema lapidescens* QL01 genomes.

Gene ID	Anotation	Identities	Source	Accession number
Gsp00921	sesquiterpene synthase	49.42%	*Collybia nuda*	BDI63095.1
Gsp01298	sesquiterpene synthase	67.85%	*Marasmiellus scandens*	KAK7454445.1
Gsp01308	sesquiterpene synthase	36.45%	*Collybia nuda*	BDI63101.1
Gsp01892	sesquiterpene synthase	32.78%	*Hypholoma fasciculare*	UPX76569.1
Gsp01900	sesquiterpene synthase	32.89%	*Hypholoma fasciculare*	UPX76569.1
Gsp02048	sesquiterpene synthase	67.46%	*Collybia nuda*	BDI63096.1
Gsp02067	sesquiterpene synthase	67.42%	*Collybia nuda*	BDI63096.1
Gsp02170	sesquiterpene synthase	70.13%	*Collybia nuda*	BDI63101.1
Gsp03073	sesquiterpene synthase	54.82%	*Collybia nuda*	BDI63100.1
Gsp03365	sesquiterpene synthase	32.79%	*Collybia nuda*	BDI63101.1
Gsp06370	sesquiterpene synthase	82.60%	*Paramarasmius palmivorus*	KAK7041430.1
Gsp06467	sesquiterpene synthase	58.72%	*Collybia nuda*	BDI63095.1
Gsp09653	sesquiterpene synthase	78.95%	*Clitopilus sp.*	BBH51500.1
Gsp10311	sesquiterpene synthase	51.38%	*Collybia nuda*	BDI63095.1
Gsp11849	sesquiterpene synthase	35.76%	*Collybia nuda*	BDO24661.1
Gsp11891	sesquiterpene synthase	36.08%	*Collybia nuda*	BDO24661.1
Gsp12017	sesquiterpene synthase	62.31%	*Pleurotus ostreatus*	BDI63110.1
Gsp00577	sesquiterpene synthase	65.54%	*Collybia nuda*	KAF9460417.1
Gsp08314	sesquiterpene synthase	51.74%	*Clitopilus sp.*	BBH51515.1

Table S17. Quantify CpG site counts per core gene.

Cluster .No	Gene name	chromosome	Core gene types	Gene start location	Gene termination location	Methylation sites
13	Gsp01766	Chr2	NRPS	6,591,072	6,638,658	6608478,6608484,6608546,6608551,6608581,6608598,6608620, 6608625,6608629,6608641,6608651,6608665,6608717,6608740, 6608768,6608813,6608857,6608863,6608892,6608900,6608946, 6608957,6609781,6609795,6609806,6609857,6609860,6609869, 6609922,6609950,6610006,6610023,6610035,6610044,6610126, 6610158,6610219,6610279,6610297,6610300,6610309,6610336, 6610339,6610384,6610391,6610522,6610566,6610641,6610666, 6610676,6610690,6610758,6610783,6610785,6610809,6610856, 6610900,6610979,6610991,6610996,6611008,6611086,6611103, 6611114,6611218,6611236,6611264,6611268,6611282,6611310, 6611337,6611342,6611349,6611428,6611439,6611457,6611468, 6611472,6611541,6611616,6611678,6611731,6611745,6611796, 6611803,6611819,6611833,6611866,6611869,6611897,6611912, 6612021,6612071,6612103,6612174,6612188,6612197,6612207, 6612209,6612242,6612245,6612304,6612311,6612315,6612350, 6612389,6612434,6612486,6612499,6612515,6612579,6612596, 6612702,6612710,6612717,6612749,6612789,6612830,6612868, 6612898,6612926,6612990,6613040,6613063,6613074,6613098, 6613164,6613188,6613271,6613279,6613308,6613319,6613359, 6613431,6613445,6613462,6613507,6613531,6613549,6613606, 6613642,6613675,6613678,6613706,6613766,6613784,6613820, 6614131,6614134,6614210,6614263,6614474,6614792,6615721, 6615762,6615805,6615843,6615847,6615887,6615899,6615958, 6615972,6615981,6615991,6615993,6616026,6616029,6616088, 6616095,6616099,6616104,6616173,6616204,6616227,6616270, 6616283,6616287,6616299,6616363,6616367,6616373,6616380, 6616396,6616401,6616416,6616489,6616497,6616518,6616576, 6616655,6616698,6616708,6616713,6616732,6616745,6616777, 6616803,6616828,6616851,6616862,6616920,6616952,6616976, 6617009,6617015,6617030,6617034,6617051,6617084,6617110, 6617119,6617147,6617183,6617207,6617261,6617327,6617339, 6617353,6617364,6617415,6617418,6617427,6617456,6617471, 6617581,6617593,6617602,6617672,6617682,6617690,6617719, 6617721,6617730,6617753,6617775,6617790,6617835,6617853, 6617892,6617938,6617940,6617947,6617980,6618065,6618069, 6618086,6618097,6618113,6618137,6618150,6618188,6618223, 6618237,6618246,6618272,6618284,6618289,6618305,6618330, 6618332,6618352,6618356,6618367,6618398,6618400,6618543, 6618555,6618557,6618566,6618591,6618595,6618602,6618608,

Cluster .No	Gene name	chromosome	Core gene types	Gene start location	Gene termination location	Methylation sites
						6618626,6618633,6618650,6618669,6618686,6618701,6618722, 6618735,6618770,6618783,6618811,6618851,6618878,6618884, 6618905,6618919,6618943,6618997,6619027,6619143,6619169, 6619184,6619251,6619255,6619278,6619282,6619318,6619324, 6619353,6619407,6619413,6619416,6619466,6619620,6619646, 6619658,6621765,6621771,6622511
21	Gsp02170	Chr3	terpene	1,803,842	1,814,061	1803849,1803918,1803962,1804070,1804101,1804168,1804183, 1804238,1804313,1804360,1804396,1804458,1804473,1804615, 1804619,1804654
29	Gsp04909	Chr5	terpene	674,185	680,885	674266,674279,674314,674318,674321,674330,674393,674463, 674476,674495,674500,674531,674552,674562,674570,674573, 674577,674610,674633,674650,674657,674672,674675,674710, 674713,674719,674725,674737,674739,674765,674794,674811, 674844,674849,674853,674859,674865,674883,674897,674943, 674946,674974,675034,675078,675081,675087,675090,675093, 675111,675147,675150
38	Gsp06593	Chr6	NRPS	4,484,271	4,526,656	4505463
42	Gsp07849	Chr7	PKS	5,762,576	5,790,301	5775521
46	Gsp08505	Chr8	PKS	4,667,825	4,708,615	4686611,4686637,4686651,4686680,4686729,4686747,4686764, 4686796,4686832,4686862,4686893,4686902,4686914,4686924, 4686953,4686968,4687061,4687065,4687068,4687070,4687100, 4687119,4687186,4687238,4687241,4687273,4687283,4687360, 4687367,4687421,4687461,4687506,4687539,4687546,4687567, 4687607,4687612,4687689,4687717,4687720,4687734,4687743, 4687877,4688060,4688561
50	Gsp09146	Chr9	PKS	2,552,571	2,584,816	2575092,2575165
53	Gsp10648	Chr10	RIPP	2,391,981	2,444,186	2414553
60	Gsp12017	Chr11	terpene	2,201,870	2,220,088	2210870,2210888,2210914,2210953,2210964,2210985,2210996, 2211010,2211017,2211044,2211071,2211080,2211094,2211145, 2211165,2211175,2211208,2211230,2211239,2211243,2211273, 2211295,2211303,2211324,2211330,2211336,2211342,2211380, 2211383,2211397,2211416,2211435,2211443,2211453,2211459, 2211485,2211499,2211505,2211521,2211527,2211581,2211601, 2211633,2211655,2211721,2211723,2211738,2211788,2211795, 2211801,2211827,2211853,2211859,2211863,2211916,2211930, 2211953,2211965

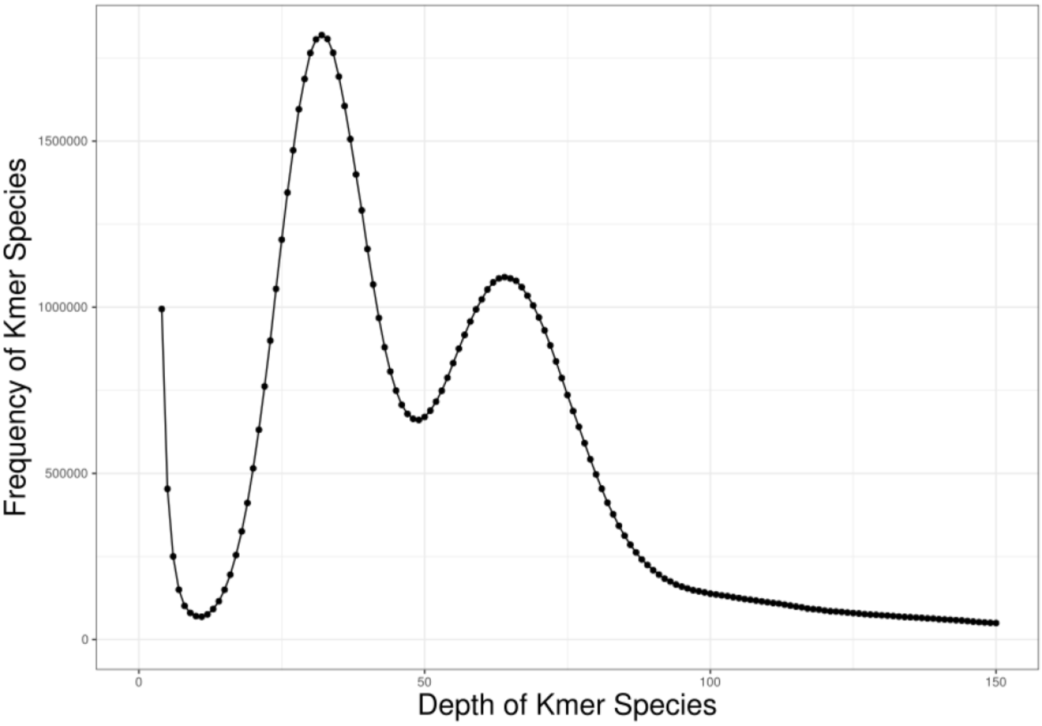

Figure S1. Kmer-Depth and Kmer Species-Frequency Distribution Plot of *Gerronema lapidescens* QL01.

The blue line represents the actual K-mer curve, the black line is the k-mer curve estimated by the model, the yellow line is the K-mer curve corresponding to the unique data, the red line represents the error curve due to sequencing errors, and the dashed line represents speculation K-mer peak.

Using the reads obtained by sequencing, K-mer-based analysis was used to estimate the genome size and heterozygosity. A K-mer refers to a sequence of K bp in length. Iteratively select a sequence of length K bases from a continuous sequence. If the length of the sequence is L and the length of the K-mer is K, then L-K+1 K-mers can be obtained. We take K-mers for the reads obtained by sequencing, and then count the frequency of each K-mer. According to the Lander waterman algorithm, the genome size (G) satisfies the following formula:

$$C_{base} = \frac{C_{k-mer} \times L}{L-K+1}$$

$$\mathrm{G} = \frac{n_{k-mer}}{c_{k-mer}} = \frac{n_{base}}{c_{base}}$$

C_{base} and $C_{k\text{-}mer}$ are the expected depth of coverage and K-mer, and n base and n k-mer are the total number of bases and the total number of K-mers in the sequence. In the case of a certain amount of data, the depth frequency of K-mer is subject to Poisson distribution, so the peak of the K-mer depth frequency distribution is the corresponding depth, which is used as an estimate of the expected depth of K-mer.

Figure S2. GC content density distribution plot of *Gerronema lapidescens* QL01.

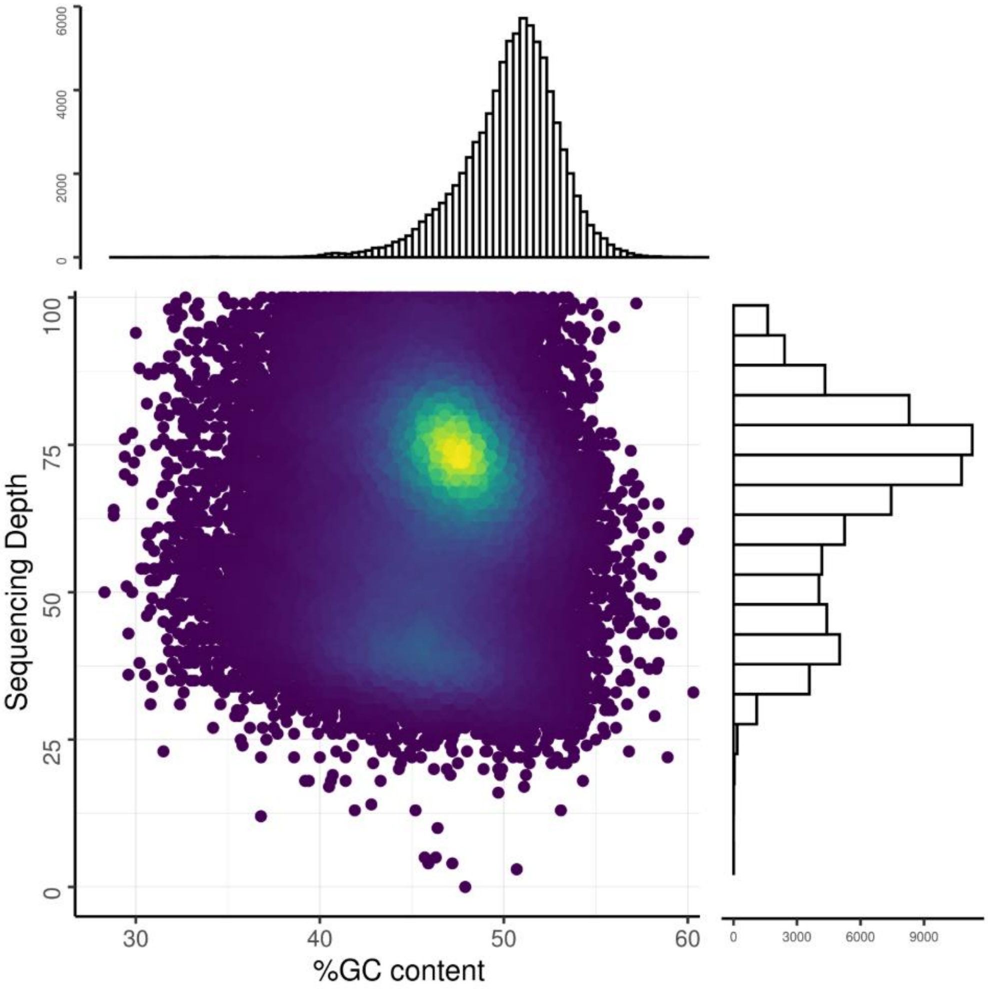

Figure S3. Sequencing depth density distribution plot of *Gerronema lapidescens* QL01.

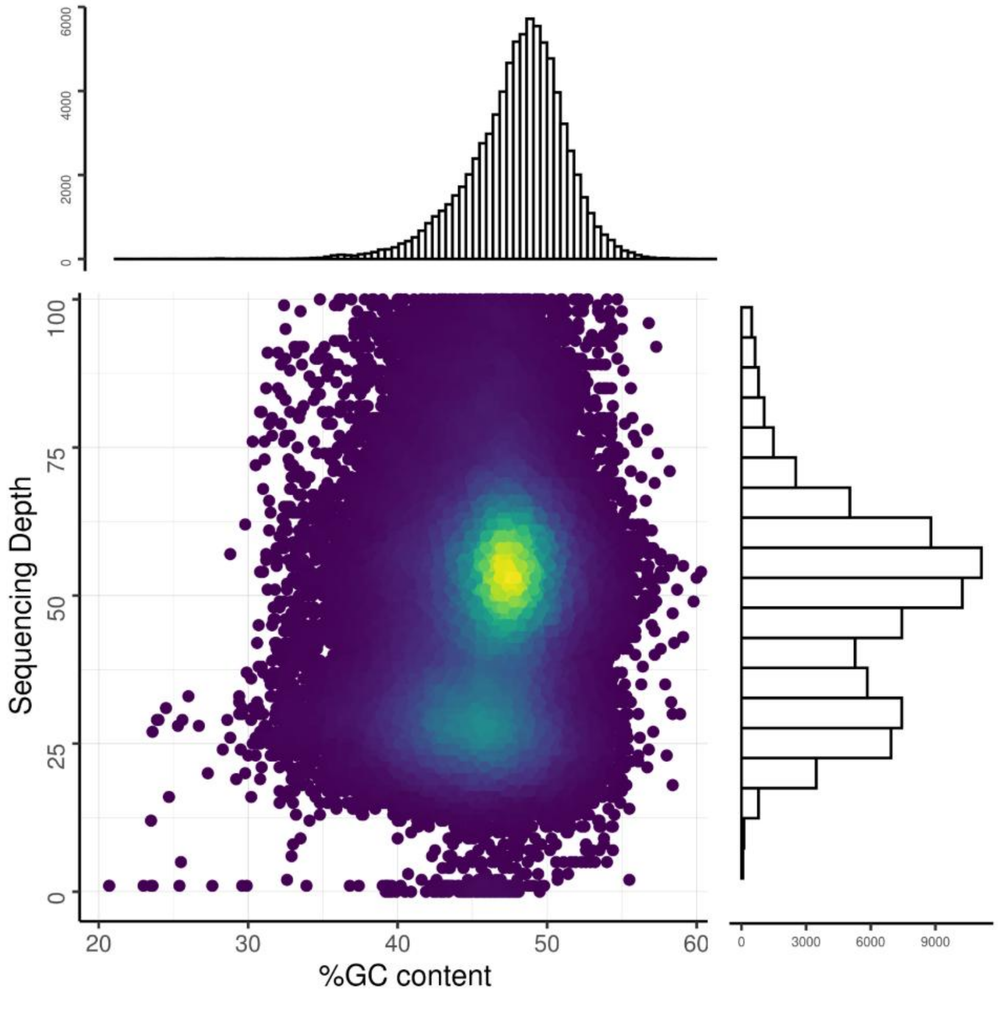

Supplement: Supplementary file 1 [file jof-11-00647-s001.zip › Supplemental Material.pdf]
